# The flexural strength of 3D-printed provisional restorations fabricated with different resins: a systematic review and meta-analysis

**DOI:** 10.1186/s12903-023-03826-x

**Published:** 2024-01-10

**Authors:** Ravinder S. Saini, Vishwanath Gurumurthy, Syed Altafuddin Quadri, Shashit Shetty Bavabeedu, Khalid M. Abdelaziz, Abdulmajeed Okshah, Abdulkhaliq Ali F. Alshadidi, Lazar Yessayan, Seyed Ali Mosaddad, Artak Heboyan

**Affiliations:** 1 Department of Dental Technology, COAMS, King Khalid University, Abha, Saudi Arabia; 2 Department of Restorative Dental Sciences, College of Dentistry, King Khalid University, Abha, Saudi Arabia; 3 Department of Therapeutic Stomatology, Faculty of Stomatology, Yerevan State Medical University after Mkhitar Heratsi, Yerevan, Armenia; 4 Student Research Committee, School of Dentistry, Shiraz University of Medical Sciences, Shiraz, Iran; 5 Department of Prosthodontics, Faculty of Stomatology, Yerevan State Medical University after Mkhitar Heratsi, Yerevan, Armenia

**Keywords:** 3-dimensional printing; dental materials, Flexural strength, Temporary dental restorations

## Abstract

**Background:**

Three-dimensional (3D) printing technology has revolutionized dentistry, particularly in fabricating provisional restorations. This systematic review and meta-analysis aimed to thoroughly evaluate the flexural strength of provisional restorations produced using 3D printing while considering the impact of different resin materials.

**Methods:**

A systematic search was conducted across major databases (ScienceDirect, PubMed, Web of Sciences, Google Scholar, and Scopus) to identify relevant studies published to date. The inclusion criteria included studies evaluating the flexural strength of 3D-printed provisional restorations using different resins. Data extraction and quality assessment were performed using the CONSORT scale, and a meta-analysis was conducted using RevMan 5.4 to pool results.

**Results:**

Of the 1914 initially identified research articles, only 13, published between January 2016 and November 2023, were included after screening. Notably, Digital Light Processing (DLP) has emerged as the predominant 3D printing technique, while stereolithography (SLA), Fused Deposition Modeling (FDM), and mono-liquid crystal displays (LCD) have also been recognized. Various printed resins have been utilized in different techniques, including acrylic, composite resins, and methacrylate oligomer-based materials. Regarding flexural strength, polymerization played a pivotal role for resins used in 3D or conventional/milled resins, revealing significant variations in the study. For instance, SLA-3D and DLP Acrylate photopolymers displayed distinct strengths, along with DLP bisacrylic, milled PMMA, and conventional PMMA. The subsequent meta-analysis indicated a significant difference in flexure strength, with a pooled Mean Difference (MD) of − 1.25 (95% CI − 16.98 - 14.47; *P* < 0.00001) and a high *I*^*2*^ value of 99%, highlighting substantial heterogeneity among the studies.

**Conclusions:**

This study provides a comprehensive overview of the flexural strength of 3D-printed provisional restorations fabricated using different resins. However, further research is recommended to explore additional factors influencing flexural strength and refine the recommendations for enhancing the performance of 3D-printed provisional restorations in clinical applications.

**Supplementary Information:**

The online version contains supplementary material available at 10.1186/s12903-023-03826-x.

## Background

The use of three-dimensional (3D)-printed temporary dental restorations is increasing in clinical settings owing to the widespread availability of intraoral scanning technology, user-friendly dental computer-aided design (CAD) software, and rapid 3D printing capabilities [1]. Recently, it has gained significant attention in the field of dentistry. It has revolutionized dental restorations, including provisional restorations [2, 3]. Utilizing technology in dental prosthesis production is more advantageous than traditional methods, such as the lost-wax technique, owing to material and energy conservation benefits, reduced carbon emissions, and cost-effectiveness [4]. Moreover, Provisional restorations rely on factors such as flexural strength to ensure that abutment teeth remain stable during the interim period [5], and they offer temporary support, protection, and aesthetics until the final restorations are made [6].

Flexural strength is the material’s resistance to bending without breaking, which is crucial for dental restorations, as it ensures that they withstand forces during mastication [7]. 3D printing technology facilitates the creation of temporary restorations using various resins, each with distinct compositions, curing procedures, and physical characteristics. These variations may influence the flexural strength of provisional restorations [8].. By evaluating the flexural strength, dental professionals can ensure the durability and longevity of restorations [9]. This assessment guides materials and fabrication techniques for optimal performance and patient satisfaction. Moreover, understanding the factors affecting flexural strength improves the design and production of 3D-printed provisional restorations, thereby enhancing clinical success rates [10].

Therefore, assessing flexural strength is crucial for evidence-based decision-making and guiding future advancements in restorative dental care. Understanding the flexural strength of 3D-printed provisional restorations using different resin materials is essential. This enables dental practitioners to make informed decisions when selecting materials with the desired mechanical properties [11]. This knowledge can help optimize the choice of resins for specific clinical scenarios, considering factors such as anticipated functional loads and occlusal forces.

Using various resins in the 3D printing of temporary restorations provides versatile possibilities and benefits [12, 13]. These temporary restorations are vital in dental practice and serve as provisional substitutes when permanent restorations are fabricated [14]. Different resins, such as methacrylate-based and photopolymerizable resins, exhibit unique properties that can be tailored to specific clinical requirements [15]. These resins differ in their mechanical strength, esthetics, biocompatibility, and ease of manipulation [16]. One important consideration when selecting resins is their flexural strength [17]. Choosing a resin with optimal flexural strength is crucial for temporary restorations to withstand occlusal forces and prevent fractures or debonding [18]. Additionally, Esthetic properties, such as color and clarity resembling natural teeth, are crucial for visually pleasing outcomes, which can enhance esthetics and patient satisfaction during the interim period [19]. Choosing biocompatible resins for temporary restorations is vital to avoid adverse reactions or complications, especially in patients who may be sensitive or allergic to specific materials [20, 21]. Efficient manipulation and rapid curing of dental materials are crucial. Quick-curing resins streamline dental workflows and reduce chairside time, improving patient comfort.

Moreover, DIN EN ISO 6872:2019 is a reference for biaxial flexural strength testing; however, additional measures are required to ensure the consistency and comparability of results across different laboratories [22]. In addition, adherence to the fabrication guidelines outlined in ISO 20795.1:2013 and ASTM D790 is recommended [23].

Conducting a systematic study and meta-analysis of the flexural strength of 3D-printed provisional restorations made from various resins is imperative for a thorough understanding of their performance and longevity. With the increasing use of 3D printing technology in dentistry, understanding the effects of different resin materials on the flexural strength of provisional restorations is crucial for clinicians and researchers. This research can aid in making informed decisions regarding material selection and treatment planning, ultimately improving the quality and longevity of dental restorations while enhancing patient care and satisfaction. Thus, the present study was designed to critically analyze and summarize the existing literature on the flexural strength of 3D-printed provisional restorations fabricated using different resins.

## Methods

This systematic review and meta-analysis adhered to the guidelines outlined by the Preferred Reporting Items for Systematic Reviews and Meta-Analysis (PRISMA) criteria [24]. The protocol used for this systematic review was the registered international platform for registered systematic reviews and meta-analysis protocols (INPLASY) (2023110054).

### Literature search

The search strategy was established according to the participants, intervention, comparators or controls, and outcome (PICO) framework [25]. Population/Participants: 3D printed provisional. Intervention: Types of resins affecting strength. Comparison or control: temporary restorations/denture bases. Outcomes: Effect of various factors on Flexural Strength. Different databases such as ScienceDirect, Web of Sciences, PubMed, GoogleScholar, and Scopus were searched using different keywords and Medical subject heading terms (MeSH) terms along with Boolean operators such as “Flexural strength [Mesh Term]” OR “Flexural” OR “Strength,” “Resistance,” “Printing, three dimensional [MeSH Term]” OR “3D printing”, “3D printing”, “CAD materials,” “Provisional restorations,” “Temporary restorations,” Interim restorations,” “Transitional restorations,“ Substitute restorations,” “Resin materials,” “polymer resins,” “Photopolymers,” “Methacrylate-based resins,” “Photopolymerizable resins,” “Ionomer” (Supplementary Table 1).

### Inclusion criteria

Studies that provided data on the flexural strength of provisional restorations made using 3D printing techniques employing various resin materials were considered. In vitro experiments, comparative studies, and clinical trials were eligible for inclusion, regardless of their location or setting. The selected studies were expected to present clear and relevant information, including the mean flexural strength values, standard deviations, and type of 3D printing technology employed. Additionally, studies incorporating resins with varying chemical compositions or characteristics, such as biocompatibility and esthetic properties, were included for comprehensive analysis and comparison. Randomized controlled trials (RCTs) and prospective or comparative studies published in peer-reviewed journals between 2013 and 2023 were included.

### Exclusion criteria

Studies lacking pertinent data on flexural strength, those not published in peer-reviewed journals, and those not presented in English were excluded. Additionally, studies involving non-human subjects or those that exclusively focused on permanent restorations rather than provisional restorations were excluded. Case series, case reports, observational studies, and reviews.

### Study selection and assessment

Original publications, study titles, and abstracts were independently evaluated. Two reviewers independently assessed the entire text of the papers that met the inclusion requirements, and their conclusions were discussed to arrive at a consensus. Any disagreements were resolved by a third independent reviewer and settled by consensus.

### Data extraction

Information retrieval was performed for the selected studies that met the inclusion criteria. After screening the papers’ titles, abstracts, and full texts, a data extraction form was used to record the extracted data. Two reviewers independently recorded each demographic characteristic (study author details, country, experimental design, and sample size), 3D Printing Parameters (printed type, risen type, layer thickness, wavelength/light intensity, temperature settings, and build orientation), control, testing method for strength, post-processing or treatment applied, conclusion, and limitations for a systematic review. The mean flexural strength values and their corresponding standard deviations are essential for statistical analysis in a meta-analysis and for constructing forest plots.

### Quality assessment

Given that all selected studies were in vitro investigations, their quality was evaluated using the CONSORT scale with 14 items (Appendix 1) for in vitro studies [26, 27].

### Data analysis

This systematic review incorporated articles through qualitative analysis. The PRISMA checklist served as the framework for systematically reviewing relevant literature, and a systematic step-by-step approach was employed to select articles. Additionally, the meta-analysis phase was conducted using RevMan 5.4 [28] to calculate the Cochrane Q and *I*^*2*^ values, quantifying trial dispersion. A random-effects model was used, with the significance level set at 0.05.

## Results

### Literature searched

**Fig. 1:**
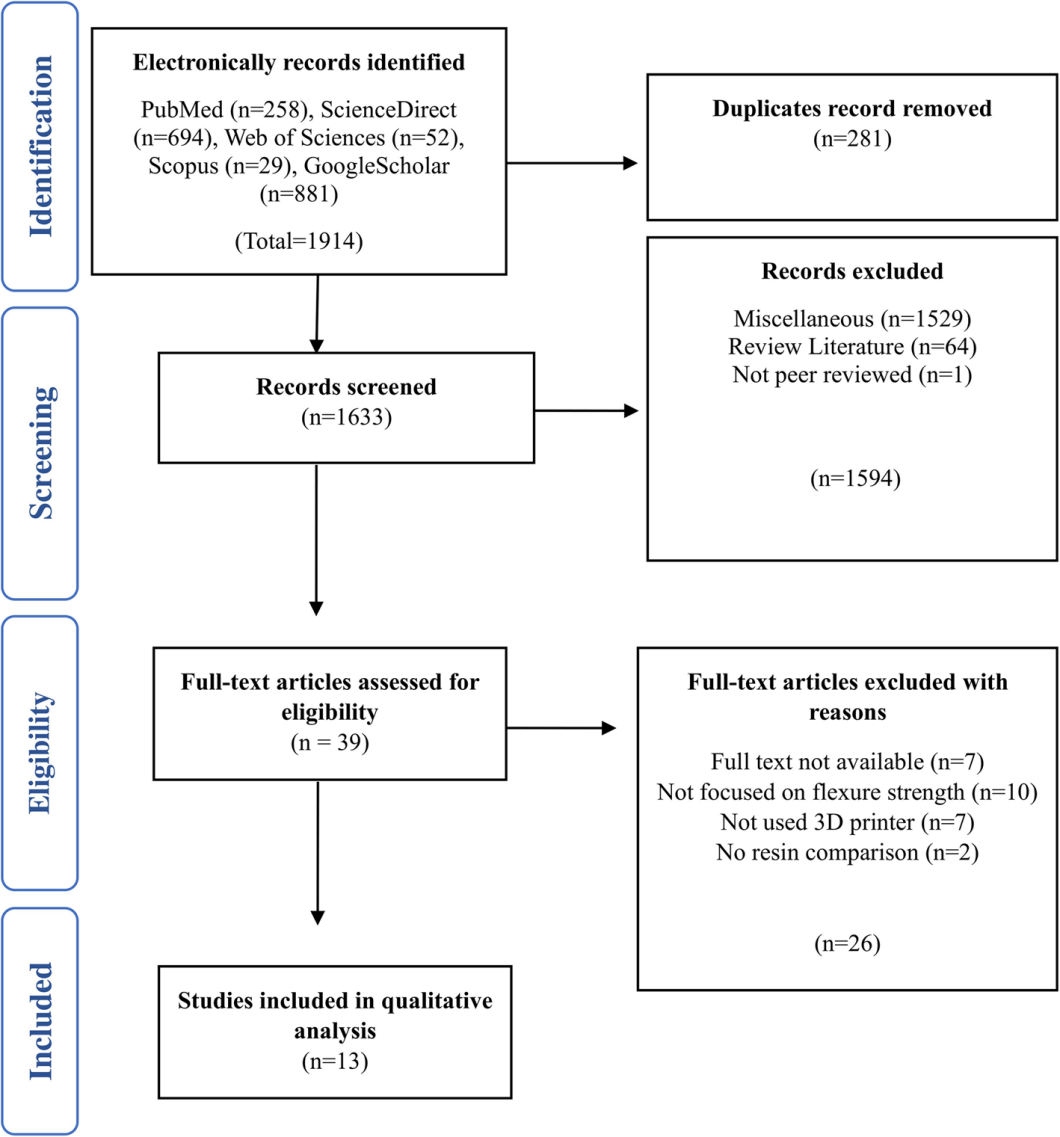
PRISMA flow chart

An exhaustive review of the scientific literature was conducted using multiple electronic databases. All the identified research articles were published in highly esteemed peer-reviewed journals. Following stringent analysis, 1914 relevant articles were identified. Subsequently, 281 duplicate articles were identified and excluded. The remaining 1633 publications underwent a meticulous examination of their titles and abstracts, which revealed that 1594 articles were not pertinent to the scope of our study and were consequently excluded. Subsequently, the remaining 39 articles were subjected to comprehensive scrutiny, resulting in the removal of 26 articles for various reasons (Fig. 1). Tables 1 and 2 have been included to provide a detailed overview of the 13 remaining studies published between January 2016 and November 2023, highlighting their essential characteristics and features.

**Table 1:** Demographic and 3D printer characteristics of the included studies

Demographic characteristics				3D Printing Parameters							
Study ID	Country	Study design	Sample size	Printed type/Model	Resin type/brand	Layer thickness	Wavelength/Light Intensity	Temperature settings	Build orientation	Post-curving time	Control
[39]	India	In-vitro	20/Each group	light-cured micro-hybrid	Envision TEC’s E-Dent 100/ GC Corporation, Japan, and Ceramill TEMP (AmannGirrbach, AG, Austria) for control.	NA	NA	NA	NA	NA	PMMA (CH) and Heat activated polymerized CH resin (CC)
[29]	S. Korea	In-vitro	5	SLA (S3Z), DLP (D3Z, D3P)	Acrylate Photopolymer, Bis-acrylic	100 μm	NA	37 °C	NA	NA	PMMA
[1]	S. Korea	In-vitro	15 specimens for each material	DLP (Nextdent Co.), SLA (Formlabs Co.), FDM (FlashForge Co.)	PMMA-based liquid photopolymer (NextDent Co., Formlabs Co., ColorFabb Co.)	DLP, SLA = 25–100 μm; FDM = 100–500 μm	NA	NA	30^0^	DLP = 120 minutes, SLA = 60 minutes	PMMA-based self-cured resin
[36]	China	In-vitro	NA	DLP, mono-LCD	Enlighten AA Temp, NextDent C&B	100 μm	NA	NA	NA	NA	NA
[34]	Saudi Arabia	In-vitro	40 acrylic specimens	DLP (ASIGA, Erfurt, Germany), SLA (Formlabs Inc., Somerville, MA, USA)	DentaBASE (ASIGA, Erfurt, Germany), Denture Base Resin LP (Formlabs Inc., Somerville, MA, USA), Denture 3D+ (NextDent B.V., Soesterberg, The Netherlands) Temporis (Composite resin) (DWS)	50 μm	DentaBASE = 405 nm/13.14 mW/cm^2^, Denture Base Resin LP = 395 nm/1.176 mW/cm^2^, Denture 3D + =405 nm/1.4 mW/cm^3^	60 °C	90^0^	Ten minutes	Heat-polymerized acrylic
[37]	Turkey	In-vitro	120	NA	Temporis (Composite resin)	60 μm	NA	5–55 °C	90^0^	NA	PMMA, Bis-acryl, CAD-CAM/Milled
[31]	Brazil	In-vitro	40/10 per group	SLA, SLS	Stratasys SLS resin, Gray Formlabs SLA resin (Restorative materials) (PA2201; Stratasys Direct Manufacturing; Gray Resin; Formlabs Inc)	NA	NA	60 °C	90^0^	NA	Acrylic resin, Bis-acryl resin
[30]	S. Korea	In-vitro	15/group	DLP (Sprintray Pro95 and NextDent 5100)/ Graphy Inc. and NextDent B.V.,	Graphy (Methacrylate oligomer derived from polyurethane resin, phosphine oxides, and pigment)/ TC-80DP/ Graphy Inc., Seoul, Korea; NextDent (> 90% methacrylate monomer, methacrylic oligomers, < 3% phosphine oxides, pigment)/ C&B MFH/ NextDent B.V., Soesterberg, The Netherlands	Grapy = 50–100 μm, NextDent = 30–100 μm	405 nm wavelength	NA	NA	30 minutes	Prefabricated zirconia crowns
[38]	Romania	In-vitro	40	DLP (printer that supports NextDent resins)	3DCS: MFH, NextDent, NextDent C;B; 3DOS: HARZ Labs Dental Sand	NA	UV-A 315–400 nm	60 °C	NA	10–12 minutes	CAP: Duracyl, CHP: Superpont C + B: SpofaDental
[32]	Brazil	In-vitro	12	SLA	Cosmos Temp	NA	NA	NA	NA	NA	Evolux PMMA, Structur 2 SC
[33]	Brazil	In-vitro	30/Each group	DLP (D30, Rapid Shape, Heimsheim, Germany)	COSMOS Temp (Yller, Pelotas, Brasil)	65 μm	NA	37 °C	NA	Seven minutes	Acrylic resin, Nanofilled composite resin, CAD/CAM PMMA resin, Bis-acryl composite resin
[35]	Saudi Arabia	In-vitro	6/Each group (6)	DLP (NextDent 5100; Nextdent, Soesterburg, the Netherlands; Asiga MAX; Asiga, Alexandria, Australia; Nova 3D Master; Nova3D, Shenzhen, China)	A1 and A2: NextDent 5100 printed with Crown & Bridge NextDent. B1 and B2: Asiga MAX printed with Asiga DentaTooth. C1 and C2 = Nova 3D Master	50 μm	NA	NA	90^0^	NA	N/A
[40]	Germany	In-vitro	50	DLP	3D-printed composite resin (VarseoSmile Crown Plus)	50 μm	NA	37 °C	90^0^	NA	Polymer-infiltrated ceramic network, Nanohybrid composite resin

*DLP* Digital Light Processing, *SLA* Stereolithography, *FDM* Fused Deposition Modeling, *SLS* Selective Laser Sintering, *PMMA* Polymethyl methacrylate, *CAD/CAM* Computer-Aided Design/Computer-Aided Manufacturing, *Mono-LCD* Monoliquid crystal display, *NA* Not available

**Table 2:** Summary of the outcomes related to flexure strength

Study ID	Testing method for strength	Flexure strength (MPa)	Post-processing or treatment applied	Conclusion	Limitations
[39]	Universal testing machine	PR = 79.54 CH = 95.58 CC = 104.20	Polymerization	Heat-activated polymerized PMMA (CH) resin (CC) had the highest flexural strength.	Study design
[29]	Universal testing machine	SLA method = 116.08, DLP Acrylate photopolymer = 46.83, DLP Bis-acrylic = 146.37, Milled PMMA = 168.57, Conventional PMMA = 89.54	NA	3D printing demonstrated clinically flexural strength to those produced using subtractive manufacturing and traditional methods	NA
[1]	Universal testing machine	Mean: DLP = 1189, SLA = 1323, FDM group did not break	NA	The flexural strength of the DLP and SLA groups was markedly more significant than the conventional group, as indicated by a statistically significant difference (*p* < 0.001)	Study design
[36]	3-point flexural bend test	> 50	Post polymerization	Enhanced effectiveness can be achieved by subjecting the printed specimens to post-polymerization in a more robust post-polymerization unit.	Flexural strength was assessed in narrower samples, adhering to the guidelines outlined by ISO standards.
[34]	3-point flexural bend test	NextDent = 56.4 Control = 93.4	Polymerization and Heat-polymerization for control resin	When compared to heat-polymerized specimens, 3D-printed specimens showed lower flexural strength	Accuracy measurement, lack of thermal, water aging
[37]	3-point flexural bend test	No significant differences.	Ultraviolet polymerization	Digitally produced intermediate materials outperformed traditionally polymerized materials in terms of mechanical characteristics	NA
[31]	3-point flexural bend test	SLA = 48.9 SLS = 77.3 Acrylic resin = 69.2 Bis-acryl resin = 75	Polymerized with light	SLS resin demonstrated positive outcomes, showcasing higher maximum flexural strength	Orientation angles and new types of resins were missing.
[30]	Universal testing machine	Graphy = 329.3 NextDent = 177.8	NA	3D-printed resin crowns might present a viable alternative for fabricating fixed prostheses for primary teeth	Study design
[38]	3-point flexural bend test	3DCS = 143 3DOS = 141 CHP = 76 CAP = 88	Polymerization	The tested 3D-printed interim resins outperformed the traditional resins	A limited number of materials were investigated and tested
[32]	3-point flexural bend test	Cosmos Temp = 56.83 Evolux PMMA = 111.76 Structur 2 SC = 87.34	post-polymerized with 3000 flashes of ultraviolet light	Although the mechanical qualities of the milled resin were more significant or comparable to those of the bisacrylic resin, the 3D-printed resin was statistically inferior to both the milled and bisacrylic resins	Orientation angles were not considered
[33]	3-point flexural bend test	3D printed = 81.33 Acrylic resin = 72.90 Nanofilled composite resin = 34.97 CAD/CAM PMMA resin = 94.63 Bis-acryl composite resin = 91.57.	NA	Except for 3D-printed resin, thermocycling lowered the flexural strength of most temporary materials	NA
[35]	3-point flexural bend test	Before accelerated aging (pre-aging), the flexural strength of the A2 group (151 ± 7) was greater (p < 0.05) than that of the other groups.	Polishing and aging	After aging, the flexural strength of the 3D-printed interim resins varied based on the material, system, and printing angle.	Study design, missing data
[40]	Piston-on-three-balls method (P3B)	3D = 83.5 Polymer-infiltrated ceramic network = 140.3 Nanohybrid composite resin = 237.3	NA	The 3D-printed composite resin exhibited the lowest mechanical properties	Staircase approach

*DLP* Digital Light Processing, *SLA* Stereolithography, *FDM* Fused Deposition Modeling, *SLS* Selective Laser Sintering, *PMMA* Polymethyl methacrylate, *CAD/CAM* Computer-Aided Design/Computer-Aided Manufacturing, *Mono-LCD* Monoliquid crystal display, *NA* Not available

### General characteristics

The studies included in the analysis were conducted in a range of countries. Most studies were conducted in South Korea [1, 29, 30] and Brazil [31–33], followed by Saudi Arabia [34, 35], China [36], Turkey [37], Romania [38], India [39], and Germany [40]. Most studies have employed Digital Light Processing (DLP) [30, 33, 35, 38, 40], stereolithography (SLA) [32], Both DLP and SLA [1, 29, 34], Fused Deposition Modeling (FDM) [1], and mono-liquid Crystal Display (LCD) [36], with a variety of printed materials, such as acrylic, composite resin, and methacrylate oligomer-based materials. The layer thickness was 50 μm in most studies [30, 34, 35, 40], and the wavelength/light intensity varied across studies, with a maximum of 405 nm/13.14 mW/cm 2[34]. The temperature settings, build orientation, and post-curing times were addressed differently (Table 1). The control materials included polymethyl methacrylate (PMMA), heat-activated polymerized Polymethyl methacrylate (PMMA) resin, self-cured resin, heat-polymerized acrylic, milled materials, acrylic resin, zirconia crowns, conventional auto-polymerized resin, conventional pressure/heat-cured acrylic resin, and various composite resins (Table 1).

### Outcomes

The study outcomes (Table 2) encompassed a comprehensive analysis of the various testing methods employed to evaluate the flexural strength of the dental materials and their respective strength values (Table 2). The most commonly used testing methods include the 3-point flexural bend test [31–38], universal testing machine [1, 29, 30, 39], and piston-on-three-ball (P3B) method [40]. Polymerization leads to flexural strengths of PR = 79.54, CH = 95.58, and CC = 104.20 [39]. In the case of the SLA-3D technique and DLP Acrylate photopolymer, flexure strengths were recorded as 116.08 and 46.83, respectively, while DLP Bis-acrylic and Milled PMMA exhibited strengths of 146.37 and 168.57, respectively, and Conventional PMMA registered 89.54 [29]. Notably, the FDM group did not experience any breakage. For values exceeding 50 MPa, post-polymerization treatment was applied to NextDent (56.4 MPa) and the control (93.4 MPa) [36]. Graphy exhibited a flexural strength of 329.3, whereas NextDent measured 177.8, with no specific treatment [30]. The flexural strengths of 3DCS, 3DOS, CHP, and CAP were 143, 141, 76, and 88, respectively [38], and they underwent polymerization. When post-polymerization was conducted with 3000 flashes of ultraviolet light, the cosmos temperature was 56.83, Evolux PMMA was 111.76, and Structur 2 SC was 87.34 [32]. Moreover, the flexural strength results before accelerated aging (pre-aging) showed that the A2 group had a significantly greater strength of 151 ± 7 MPa (*p* < 0.05) than the other groups following the polishing and aging procedures [35]. The flexural strengths of the 3D polymer-infiltrated ceramic network and nanohybrid composite resin were 83.5, 140.3, and 237.3, respectively [40]. Most studies reported that the study design was a limitation (Table 2).

### Meta-analysis

**Fig. 2:**
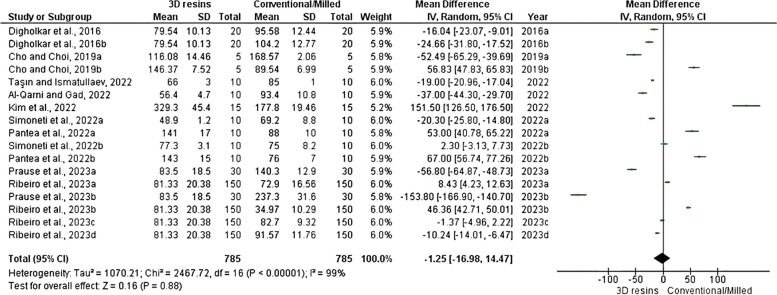
Forest plot for flexure strength

Nine studies with 785 samples in the intervention (3D printed) and control groups (resin used in conventional or milled techniques) were included to assess the flexural strength of 3D-printed provisional restorations fabricated with different resins. As shown in Fig. 2, our pooled result found a significant difference in flexure strength, with a pooled Mean Difference (MD) of − 1.25 (95% CI − 16.98 - 14.47; *P* < 0.00001) and *I*^*2*^ = 99%.

### Quality assessment

All studies (13) included the abstract, introduction, intervention, outcome, statistical method, and results (Items 1–4, 10, and 11) [1, 29–40]. While 12 studies delved into the limitations of the trials (Item 12), nine disclosed information about their funding sources (Item 13) [1, 30–40]. Interestingly, none of the studies addressed sample size calculation for the specimens (Item 5) or accessibility of the full trial protocol (Item 14). Additionally, there was a notable absence of information regarding the method used to generate a random allocation sequence (item 6) in any of the studies. Furthermore, none of the studies provided details regarding the blinding of the examiners or information about the researcher responsible for generating the random allocation (Items 8 and 9), as outlined in Table 3.

**Table 3:** Quality assessment of In-vitro studies

Studies	Item														
	1	2a	2b	3	4	5	6	7	8	9	10	11	12	13	14
[39]	Y	Y	Y	Y	Y	N	N	N	N	N	Y	Y	Y	Y	N
[29]	Y	Y	Y	Y	Y	N	N	N	N	N	Y	N	N	N	N
[1]	Y	Y	Y	Y	Y	N	N	N	N	N	Y	Y	Y	Y	N
[36]	Y	Y	Y	Y	Y	N	N	N	N	N	Y	Y	Y	N	N
[34]	Y	Y	Y	Y	Y	N	N	N	N	N	Y	Y	Y	Y	Y
[37]	Y	Y	Y	Y	Y	Y	N	N	N	N	Y	Y	Y	N	N
[31]	Y	Y	Y	Y	Y	N	N	N	N	N	Y	Y	Y	Y	N
[30]	Y	Y	Y	Y	Y	N	N	N	N	N	Y	Y	Y	Y	N
[38]	Y	Y	Y	Y	Y	N	N	N	N	N	Y	Y	Y	Y	Y
[32]	Y	Y	Y	Y	Y	N	N	N	N	N	Y	Y	Y	N	N
[33]	Y	Y	Y	Y	Y	N	N	N	N	N	Y	Y	Y	Y	N
[35]	Y	Y	Y	Y	Y	N	N	N	N	N	Y	Y	Y	Y	N
[40]	Y	Y	Y	Y	Y	N	N	N	N	N	Y	Y	Y	Y	N

*Y* Yes, *N* No

## Discussion

The flexural strength of 3D-printed provisional restorations is critical for assessing their structural integrity and suitability for clinical use [41]. As digital technologies continue to reshape the landscape of prosthodontics, the choice of printing materials plays a pivotal role in determining the mechanical performance of the final restorations [42]. This study investigated the flexural strength of 3D-printed provisional restorations, focusing on the influence of different resin materials. By scrutinizing the mechanical properties of these restorations, we aimed to provide valuable insights that can inform clinicians and researchers about the comparative strengths associated with various resin options, ultimately guiding informed decision-making in the realm of digitally fabricated provisional prosthetics.

In the present study, DLP was the most commonly used 3D technique, which may be due to its efficiency, speed, and high-resolution capabilities [43]. In dental applications, where precision and quick turnaround times are paramount, DLP technology excels by utilizing a digital light source to selectively cure all layers of liquid resin simultaneously [44]. This simultaneous curing accelerates the printing process compared to other methods, such as LCD 3D, SLA, or FDM [45]. In addition, DLP printers often provide a higher resolution, enabling the production of intricately detailed dental structures with exceptional accuracy [46]. The ability to rapidly produce precise, high-quality dental models and prosthetics has positioned DLP as the preferred choice, streamlining the workflow in dental laboratories and clinics [46]. Meanwhile, a statistically significant difference in trueness was observed when comparing the LCD 3D printer and DLP 3D printers (*p* = 0.004). Similarly, for precision, a statistically significant difference was found between the LCD 3D printer and DLP 3D printers (*p* = 0.011), indicating that the DLP 3D printer exhibited greater accuracy in dental model printing than the LCD 3D printer [47]. Similarly, no statistically significant differences were observed among the four software types analyzed using the DLP printer. Nevertheless, a group comprising the amalgamation of D-CAD (Blender–InLAB) exhibited the highest average (− 0.0324 SD = 0.0456), demonstrating superior accuracy compared to the group with the lowest average (consisting of the Meshmixer and Blender models), which included generic and specific software (0.1024 SD = 0.0819) [48]. Furthermore, DLP printers showed a notable advantage over LCD printers in another study, displaying lower RMS values and less shrinkage in 5-unit and full-arch cases. Point deviation analysis revealed significant directional differences in all DLP-printed restorations. However, only a few LCD printing and DLP printer cases have proven to be the most accurate for short-unit restorations, demonstrating reduced deviation and shrinkage [49]. In contrast, the DLP and FDM groups observed significant differences in trueness and precision. The average trueness values for DLP and FDM were 0.096 (0.021) (*P* < 0.001) and 0.063 mm (0.024) (P < 0.001), respectively. Similarly, the average precisions for DLP and FDM were 0.027 mm (0.003) (P < 0.001) and 0.036 mm (0.003) (P < 0.001), respectively. Notably, widening (0.158 mm [0.089] for DLP and 0.093 mm [0.005] for FDM, *P* = 0.05) and twisting (0.03 mm [0.014] for DLP and 0.043 mm [0.029] for FDM, P = 0.05) of the printed models were observed. FDM demonstrated greater accuracy, suggesting its suitability as a viable alternative to DLP [50].

Moreover, various printed materials, such as acrylic, composite resin, and methacrylate oligomer-based materials, have been identified. These diverse substances cater to different applications and offer a range of properties, including strength, flexibility, and biocompatibility. Acrylic polymers, known for their durability and versatility, are commonly utilized in 3D printing because of their adaptability to various applications [51]. Composite resins blend different materials for enhanced characteristics, balanced strength, and aesthetics, making them suitable for dental and aesthetic applications [52]. With their unique chemical compositions, methacrylate oligomer-based materials contribute to developing materials with specific properties often used to synthesize resins optimized for 3D printing processes [53]. The utilization of these materials underscores the flexibility of 3D printing technologies in accommodating a wide array of applications and functional requirements.

Polymerization plays a crucial role in determining the flexural strengths of different materials, and notable variations were observed in this study. For instance, SLA-3D and DLP Acrylate photopolymers exhibited distinct strengths, as did DLP bisacrylic, milled PMMA, and conventional PMMA. These diverse findings underscore the complex interplay of material composition, printing techniques, and post-processing treatments in determining flexural strength (Table 2). The question arises as to why polymerization plays a vital role because it is a chemical process by which monomers, the building blocks of polymers, join together to form a larger, more complex structure. In 3D printing, this process is fundamental for creating solid and durable objects from liquid or semi-liquid resin materials [54]. The resin transforms from a liquid or semi-liquid state to a solid state during polymerization, creating a three-dimensional network of polymer chains. The polymerization process’s extent and efficiency directly affect the printed object’s final mechanical properties, including its flexural strength [55, 56]. Incomplete polymerization can result in structural weaknesses, reduced bond strength between polymer chains, and compromised mechanical properties. In contrast, well-controlled and thorough polymerization contributed to forming a robust and homogenous material, enhancing its flexural strength. As shown in Table 2, the different resin materials used in the 3D printers underwent polymerization. This underscores the significance of the polymerization process in influencing the mechanical properties, particularly the flexural strength of 3D-printed resin materials [57]. Similarly, in another study, 40 resin samples were mechanically tested using a universal testing machine, with subsequent fractographic analysis of the failed bending samples. Additively manufactured samples demonstrated higher elastic moduli (2.4 ± 0.02 GPa and 2.6 ± 0.18 GPa) and average bending strength (141 ± 17 MPa and 143 ± 15 MPa) compared to conventional samples (1.3 ± 0.19 GPa and 1.3 ± 0.38 GPa for elastic moduli; 88 ± 10 MPa and 76 ± 7 MPa for bending strength). The results indicated greater homogeneity in the materials produced through additive manufacturing [38]. In contrast, different materials were polymerized in another study, and the flexural strength, including cold-polymerized PMMA, recorded 125.90 MPa for heat-polymerized PMMA, 140 MPa for auto-polymerized bis-acryl composite (133 MPa), and light-polymerized urethane dimethacrylate resin measured 80.84 MPa. Notably, the highest flexural strength was observed for heat-polymerized PMMA. The lightly polymerized urethane dimethacrylate resin exhibited the lowest flexural strength, indicating a significant difference in strength between the materials [5].

The meta-analysis in the present study indicated a statistically significant difference in flexure strength between the groups (MD = − 1.25, 95% CI: − 16.98 - 14.47, *p* < 0.00001). An *I*^*2*^ value of 99% suggested high heterogeneity among the included studies, indicating substantial variability in effect sizes. A negative mean difference shows reduced flexure strength in the experimental group compared to the control group. This may be due to differences in the characteristics of the compared groups, diverse methodologies for assessing flexural strength, and disparities in the types of materials or 3D printing technologies employed. The sample size and geographic location might have also contributed to the observed heterogeneity. Our findings align with those of other studies, and milling techniques demonstrated notably higher flexural strength values (Hedge g = − 3.88; 95% CI, − 7.20 to − 0.58; *P* = 0.02), and this difference persisted even after the aging process (Hedge g = − 3.29; 95% CI, − 6.41 to − 0.17; *P* = 0.04) compared to printing [58]. Similarly, the milled resin exhibited mechanical properties in flexure strength that were superior or comparable to those of the bisacrylic resin. In contrast, 3D-printed resins demonstrate statistically inferior properties compared to milled and bisacrylic resins [32]. In contrast, one study indicated that printed samples exhibited higher mean bending strengths (141 ± 17 and 143 ± 15 MPa) than traditional samples (88 ± 10 and 76 ± 7 MPa). These findings suggest superior mechanical properties in terms of elastic moduli and bending strength for printed samples and imply a higher degree of homogeneity in the material when produced through printing processes [38]. The mean flexural strengths for CAD/CAM, injection molding, and compression molding were 97.46, 84.42, and 71.72, respectively, with corresponding standard deviations of 9.93, 10.42, and 11.58, respectively. Statistical analysis indicated that CAD/CAM is the optimal denture fabrication method, exhibiting the highest mean flexural strength and lowest standard deviation compared to compression and injection molding [59].

Although this study offers valuable insights, its strengths and limitations should be acknowledged. The strength lies in the comprehensive synthesis of existing literature, which provides a collective understanding of the flexural strength across various 3D printing resins. Meta-analysis adds quantitative rigor to the findings, thus enhancing their statistical robustness. However, the limitations include potential heterogeneity among the included studies arising from variations in methodologies, printing technologies, and materials. Reliance on available published data may introduce publication bias, and the dynamic nature of 3D printing technologies may result in temporal discrepancies. Despite these limitations, this study is valuable for clinicians, researchers, and industry professionals seeking evidence-based insights into the flexural strength of 3D-printed provisional restorations.

## Conclusions

This systematic review and meta-analysis comprehensively examined the flexural strength of 3D-printed provisional restorations crafted using diverse resins. The pooled results revealed a significant difference in the flexural strength between the studied resin materials, emphasizing careful consideration when selecting materials for provisional restorations. Notably, the heterogeneity observed in the meta-analysis underscores the variability in methodologies and material characteristics across the included studies. However, the negative mean difference suggests a lower flexural strength in certain experimental groups than in the controls. Further research and subgroup analyses are imperative to unravel the sources of heterogeneity and refine our understanding of the nuanced factors influencing the flexural strength of 3D-printed provisional restorations with different resin compositions.

### Supplementary Information

**Additional file 1:  Appendix 1. **List of Items (CONSORT Scale).

**Additional file 2: Supplementary Table 1. **Literature search strategy.

## Data Availability

The data supporting this study’s findings are available from the corresponding author upon reasonable request.

## Abbreviations

3D: Three-dimensional
DLP: Digital Light Processing
SLA: Stereolithography
FDM: Fused Deposition Modeling
LCD: Liquid Crystal Display
MD: Mean difference
CAD: Computer-aided design
RCTs: Randomized controlled trials
PMMA: Polymethyl methacrylate
P3B: Piston-on-three-ball method
