# Supplementary material for: The flexural strength of 3D-printed provisional restorations fabricated with different resins: a systematic review and meta-analysis
Source: BMC Oral Health. 2024 Jan 10;24:66. doi: 10.1186/s12903-023-03826-x (PMC10782672; doi:10.1186/s12903-023-03826-x)
Supplement: Supplementary file 1 — Additional file 1: Appendix 1. List of Items (CONSORT Scale). [file 12903_2023_3826_MOESM1_ESM.docx]

**Appendix 1 List of Items (CONSORT Scale)**

Item 1: Structured abstract.

Items 2a and 2b are related to the introduction:

Item 2a: scientific background and rational explanation.

Item 2b: Introduction should have specific objectives and hypotheses).

Items 3 to 10 are related to Methodology:

Item 3: Intervention for each group.

Item 4: Completely defined, pre-specified primary and secondary measures of outcome.

Item 5: Sample size determination.

Item 6: Method used to generate the random allocation sequence.

Item 7: Mechanism used to implement the random allocation sequence.

Item 8: Who generated the random allocation sequence.

Item 9: If done, who was blinded after assignment to intervention and how.

Item 10: Statistical methods used to compare groups for primary and secondary outcomes.

Item 11: For each primary and secondary outcome, results for each group and the estimated size of the effect and its precision (for example 95% confidence interval).

Item 12: Trial limitations.

Item 13: Sources of funding and other support, the role of funders.

Item 14: Where the full trial protocol can be accessed.
