# Supplementary material for: The flexural strength of 3D-printed provisional restorations fabricated with different resins: a systematic review and meta-analysis
Source: BMC Oral Health. 2024 Jan 10;24:66. doi: 10.1186/s12903-023-03826-x (PMC10782672; doi:10.1186/s12903-023-03826-x)
Supplement: Supplementary file 2 — Additional file 2: Supplementary Table 1. Literature search strategy. [file 12903_2023_3826_MOESM2_ESM.docx]

| **Databases** | **Search terms** |
| --- | --- |
| PubMed | ((((Flexural Strength) OR (Flexural resistance)) AND ((((3D printed) OR (3D printing)) OR (Computer-aided design materials)) OR (CAD materials))) AND (((((Provisional Restorations) OR (Temporary restorations)) OR (Interim restorations)) OR (Transitional restorations)) OR (Substitute restorations))) AND ((((((Resins materials) OR (Polymer resins)) OR (Photopolymers)) OR (Methacrylate-based)) OR (Photopolymerizable)) OR (Ionomer)) |
|  | (((((3D printed) OR (3D printing)) OR (Computer-aided design materials)) OR (CAD materials)) AND (((((Provisional Restorations) OR (Temporary restorations)) OR (Interim restorations)) OR (Transitional restorations)) OR (Substitute restorations))) AND ((((((Resins materials) OR (Polymer resins)) OR (Photopolymers)) OR (Methacrylate-based)) OR (Photopolymerizable)) OR (Ionomer)) |
| ScienceDirect | (3D printed OR 3D printing OR CAD materials) AND (Provisional Restorations OR Temporary restorations OR Interim restorations) AND (Resins materials) |
| Google Scholar | (Flexural Strength OR Flexural resistance) AND (3D printed OR 3D printing OR Computer-aided design materials OR CAD materials) AND (Provisional Restorations OR Temporary restorations OR Interim restorations OR Transitional restorations OR Substitute restorations) AND (Resins materials OR Polymer resins OR Photopolymers OR Methacrylate-based OR Photopolymerizable OR Ionomer) |
| Web of Sciences | (Flexural Strength OR Flexural resistance) AND (3D printed OR 3D printing OR Computer-aided design materials OR CAD materials) AND (Provisional Restorations OR Temporary restorations OR Interim restorations OR Transitional restorations OR Substitute restorations) AND (Resins materials OR Polymer resins OR Photopolymers OR Methacrylate-based OR Photopolymerizable OR Ionomer) |
| Scopus | (3D printed OR 3D printing OR Computer-aided design materials OR CAD materials) AND (Provisional Restorations OR Temporary restorations OR Interim restorations OR Transitional restorations OR Substitute restorations) AND (Resins materials OR Polymer resins OR Photopolymers OR Methacrylate-based OR Photopolymerizable OR Ionomer) |

**Supplementary Table 1. Literature search strategy.**
